# Detection of oral potentially malignant disorders and oral cancer with white light through deep learning: a systematic review and meta-analysis

**DOI:** 10.1007/s00784-026-07058-5

**Published:** 2026-08-26

**Authors:** N. Soto Ayén, J. A. Rodríguez Molinero, P. J. Navarro Lorente, Juan Antonio Ruiz-Roca, P. López-Jornet

**Affiliations:** 1 Department of Dermatology, Stomatology, Radiology and Physical Medicine, Faculty of Medicine, University of Murcia, Murcia, Spain; 2 Faculty of Dentistry, University Rey Juan Carlos (Madrid), Madrid, Spain; 3 Artificial Intelligence, Information and Communications Technology Department, Polytechnic University of Cartagena (UPCT), Cartagena, Spain; 4 University Institute for Research in Aging-University of Murcia, Murcia, Spain; 5 Hospital Morales Meseguer, Clínica Odontológica Universitaria, 2nd floor, Avda. Marqués de los Vélez s/n, Murcia, 30008 Spain

**Keywords:** Oral potentially malignant disorders, Oral cancer, Deep learning, AI algorithms white light

## Abstract

**Objectives:**

The research question was: How accurate is artificial intelligence (AI) in diagnosing Oral Potentially Malignant Disorders (OPMD)/oral cancer in patients of any age, using digital photographic images under white light?

**Materials and methods:**

A systematic review was performed in the PubMed, Web of Science and Scopus databases. The inclusion criteria were: detection of OPMD or Oral Squamous Cell Carcinoma (OSCC). The risk of bias was assessed with the QUADAS-C tool. Forest plots were generated with the specificity and sensitivity of oral cancer and OPMD. A bivariate random effects meta-analysis was performed, by combining sensitivity and specificity.

**Results:**

The bivariate model showed a pooled sensitivity of 0.81 (95% CI, 0.728-0872) and a grouped specificity of 0.161 (95% CI, 0.113–0.223) for OPMD detection, and a combined sensitivity of 0.836 (95% CI, 9.730–0.906) and combined specificity of 0.138 (95% CI, 0.081–0.226) for oral cancer.

**Conclusions:**

Despite substantial heterogeneity among the various Deep Learning algorithms evaluated, most models demonstrated acceptable diagnostic capability for identifying OPMD and OSCC from digital images. These findings support the promising role of artificial intelligence in enhancing diagnostic processes within dentistry.

**Clinical relevance:**

Being able to diagnose OPMD through the use of digital photography allows for an early diagnosis, especially in places with limited resources. The possibility of performing assessments via smartphone devices increases accessibility and reduces dependence on costly specialized equipment, broadening its applicability in clinical practice.

**Supplementary Information:**

The online version contains supplementary material available at https://doi.org/10.1007/s00784-026-07058-5.

## Introduction

Head and neck cancer is the sixth most common malignancy worldwide, with Oral Squamous Cell Carcinoma (OSCC) accounting for approximately 90% of oral cancers [1–3]. OSCC most frequently affects the lateral tongue borders and floor mouth [4, 5]. Oral carcinogenesis is multifactorial and has been associated with risk factors including tobacco and alcohol consumption, human papillomavirus infection, sun exposure, nutritional habits, and chronic inflammatory or traumatic oral conditions [3, 6–15].

Most patients who develop OSCC present a previous history of oral potentially malignant disorders (OPMDs) [2]. According to the WHO 2020 classification, OPMDs include leukoplakia, erythroplakia, proliferative verrucous leukoplakia, erythroleukoplakia, oral lichen planus, lichenoid oral lesions, graft-versus-host disease, discoid lupus erythematosus, submucosal oral fibrosis, epidermolysis bullosa, dyskeratosis congenita, actinic cheilitis, and reverse smoking lesions of the palate [16–20].

OSCC is frequently diagnosed at advanced stages, with the 5-year survival rate remains close to 50% [2, 8, 21]. This highlights the importance of further investigating strategies and biological mechanism that facilitate earlier detection [2, 7, 8, 21–23], thereby contributing to improve patient outcomes [11, 15]. Although histopathological evaluation of a biopsy specimen- preceded by conventional clinical assessment under white light with palpation and, when indicated, cytology- continues to represent the diagnostic gold standard, a variety of adjunctive techniques have been introduced to complement routine examination. These include vital staining methods such as toluidine blue and Lugol’s iodine, light-based technologies like autofluorescence (VELscope^®^, Identafi 3000^®^, EVINCE^®^ and GOCCLES^®^), as well as narrow-band imaging and chemiluminescence [24–27].

Despite their supportive role in clinical assessment, these adjunctive modalities are not exempt from limitations, particularly in terms of cost-effectiveness and accessibility, which may restrict their integration into routine practice [2]. More recently, non-invasive diagnostic approaches have incorporated imaging-based techniques, including standardized white-light digital imaging and spectrophotometric evaluation, especially within screening protocols for oral potentially malignant disorders (OPMD) and oral cancer [28]. Simultaneously, there has been a notable expansion in studies investigating AI-driven algorithms for diagnostic and prognostic in OSCC and OPMD [10, 29–32]. Although AI applications do not currently replace histopathological confirmation or conventional examination, they may enhance diagnostic accuracy and support clinical decision-making [33].

AI refers to a broad spectrum of computational approaches designed to perform functions that traditionally required human cognitive abilities [34], enabling machines to learn patterns and execute tasks in a manner comparable to human reasoning. Within this domain, “Computer vision” is the AI discipline that focuses specifically on the processing and interpretation of visual data, constituting the foundation of contemporary image analysis. Progress in the area has been largely driven by developments in “machine learning” and, more recently, “deep learning” techniques [2, 34]. Deep learning has replaced conventional machine learning as the gold standard in image analysis [35–38], owing to their capacity to autonomously extract hierarchical and increasingly complex features from visual inputs [10, 39].

Deep learning has emerged as a central methodology in image-driven diagnostic research and has demonstrated robust performance in the detection of multiple malignancies, including breast, lung, skin and of course, oral cancers [8, 37, 40–42]. Among its medical images processes that are essential for lesion identification, boundary delineation, and quantitative assessment, these capabilities have positioned deep learning models as key tools in contemporary medical image analysis and oncologic research [7, 10, 36, 41, 43–45].

The introduction of these learning methods to identify patterns in data from patients with OPMD/oral cancer for the prediction of results in still in its nascent stages as compared with other malignant neoplasms [30, 46]. Despite growing interest, this field is still in an early stage of clinical translation. Furthermore, the integration of automated diagnostic systems holds particular relevance in low-resource environments, where access to experienced clinicians and specialized care may be limited. In such contexts, these technologies may contribute not only to expanding diagnostic coverage but also to reducing interobserver variability, as well as to be able to eliminate the subjective portion of the clinician during diagnosis [23, 40, 44, 47, 48].

Most previous studies have thoroughly examined the application of AI in diagnosing primary malignant tumors (PMTs)/cancer under different types of light, but our work focuses on images taken under white light, as this is the light routinely used in all clinics and the most accessible to professionals. Therefore, applying AI to this type of image could be of great importance for its use in all kinds of low-budget settings.

Thus, assuming that different AI models could identify visual patterns specific to OPMD/oral cancer, just as well as a human expert, the aim of the present work was to analyze and assess the use of artificial intelligence as a complement to conventional methods for the early diagnosis of OPMD/oral cancer through digital photographic images taken under white light.

## Materials and methods

A systematic review was performed according to the PRISMA-DTA (Preferred Reporting Item for Systematic reviews and Meta-Analyses Diagnostic Test Accuracy) checklist [49], and after obtaining a record at PROSPERO (International Prospective Register of Systematic Reviews) (ID: CRD42024629226).

To this end, a PICO question was defined, which consisted of: What is the accuracy of Deep learning to diagnose OPMD/oral cancer in patients of any age, through digital photographic images?, where: Participants (P): patients of any age with a diagnosis of OPMD/oral cancer; Intervention (I): use of deep learning to diagnose OPMD/oral cancer through digital photographic images; Comparison (C): no control group was defined; Outcomes (O): accuracy of Deep learning to diagnose OPMD or oral cancer through digital photographic images.

### Search strategy and study selection

The systematic review and meta-analysis was conducted by searching the main biomedical databases such as Pubmed (Medline), Web of Science, and Scopus, delimited between November, 2000, to November, 2025, aside from a search in different physical journals and books known as grey literature.

The title, abstract, and key words were searched using free terms and combined thesaurus terms from different databases, including those found in Medical Subject Headings (MeSH) and Embase Subject Headings (Emtree) to maximize sensitivity (Table 1 in supplementary material). Two authors (NSA and JARR) extracted the data from the selected studies, filling a standardized data collection form using Excel and Word (v.16/2018, Microsoft, Redmond, WA, USA). When discrepancies appeared, a third author (JRM) intervened to come to a consensus. For this, and to ensure criteria equality, inter-operator agreement tests were performed, obtaining a Cohen’s Kappa of K = 0.94, which indicates similarity in the eligibility criteria of each operator.

### Inclusion criteria

The inclusion criteria for the systematic review were: (1) articles written in English, French, Italian, Portuguese, or Spanish (2) original articles, clinical trials, observational studies; (3) studies that included the clinical diagnosis of OPMD (leukoplakia, proliferative verrucous leukoplakia, erythroplakia, oral submucosal fibrosis, oral lichen planus, actinic cheilitis, inverted palate lesions in smokers, oral lupus erythematosus, dyskeratosis congenita, oral lichenoid lesions, and graft-versus-host disease in the oral cavity), or oral cancer, oral squamous cell carcinoma, head and neck cancer; (4) original and complete research articles; (5) studies that used AI algorithms of any kind to provide diagnostic information about OPMD/oral cancer; (6) prospective or retrospective design.

The criterion for performing the meta-analysis was that the studies must include performance indices of the Deep Learning techniques applied for the prediction, such as sensitivity, specificity, true positive, false positive, false negative, true negative, positive predictive value (PPV), negative predictive value (NPV), F1 score or other metrics, which allow estimating the precision of the model.

The exclusion criteria were: (1) Letters to the editor, case reports, reviews (scoping review, systematic reviews, meta-analysis), in-vitro studies, studies with animals, meetings, and comments; (2) Insufficient availability of AI diagnostic data; (3) Application of machine learning or deep learning on any other type of photographic image that was not an image taken under white light.

### Data extraction and synthesis

The following data were extracted from each included study: year and country where the study took place, type of study, type of Deep learning architecture or model, best results, number of participants, number of images obtained, type of image, type of tool used for taking the image, histological control, location of the lesion in the oral cavity, true positive, false positive, true negative, false negative, sensitivity, specificity, mean, positive predictive value, negative predictive value, area under the curve (AUC), pixels, and region of interest (ROI)/whole image.

### AI task categorization

The included deep learning models were categorized according to their primary computer vision task into: (1) image classification models, which assigned diagnostic labels to whole images or predefined regions of interest, and (2) object detection models, which simultaneously localized and classified lesions within clinical photographs using bounding boxes or detection frameworks.

### Risk of bias assessment

The risk of bias of the studies included was assessed with the QUADAS-C (Quality Assessment of Diagnostic Accuracy Studies-Comparative) tool, which was developed as an extension of the QUADAS-2 tool to assess the risk of bias in comparative studies on the accuracy of diagnostic tests. The QUADAS-C tool maintains the same structure of 4 domains of the QUADAS-2 tool (Patient selection, Index test, Reference standard, and Flow and timing) and is comprised of additional questions to each of the QUADAS-2 domains. According to the score obtained in these domains, the risk of bias is considered “low”, “high”, “not clear” [50, 51].

A risk of bias assessment for comparative accuracy requires a bias risk assessment for the accuracy of each test (resulting from QUADAS-2) and additional criteria specific to test comparisons. The QUADAS-C tool will be useful for the systematic reviews of the exactness of the diagnostic test that address comparative issues. In addition, the researchers can use this tool to identify and avoid risk of bias when designing a comparative study of the exactness of diagnostic tests.

This risk of bias assessment was performed by two experienced researchers (JARR and JARM). The discrepancies were resolved through discussion and mutual agreement between the authors.

### Statistical analysis

Only studies with sufficiently homogeneous data were included for a valid comparison to be possible. The analysis was organized into two groups according to the main objective: on the one hand, the studies that used Deep learning models to assess OPMD, and on the other, those that were centered on the diagnosis of oral cancer.

For this, a bivariate random effects meta-analysis was performed (Reitsma model), by combining sensitivity and specificity of 21 studies with the use of the R software, v. 4.5.2, and packages meta 8.2-1, and meta 0.5.12.

## Results

### Database search results

**Fig. 1:**
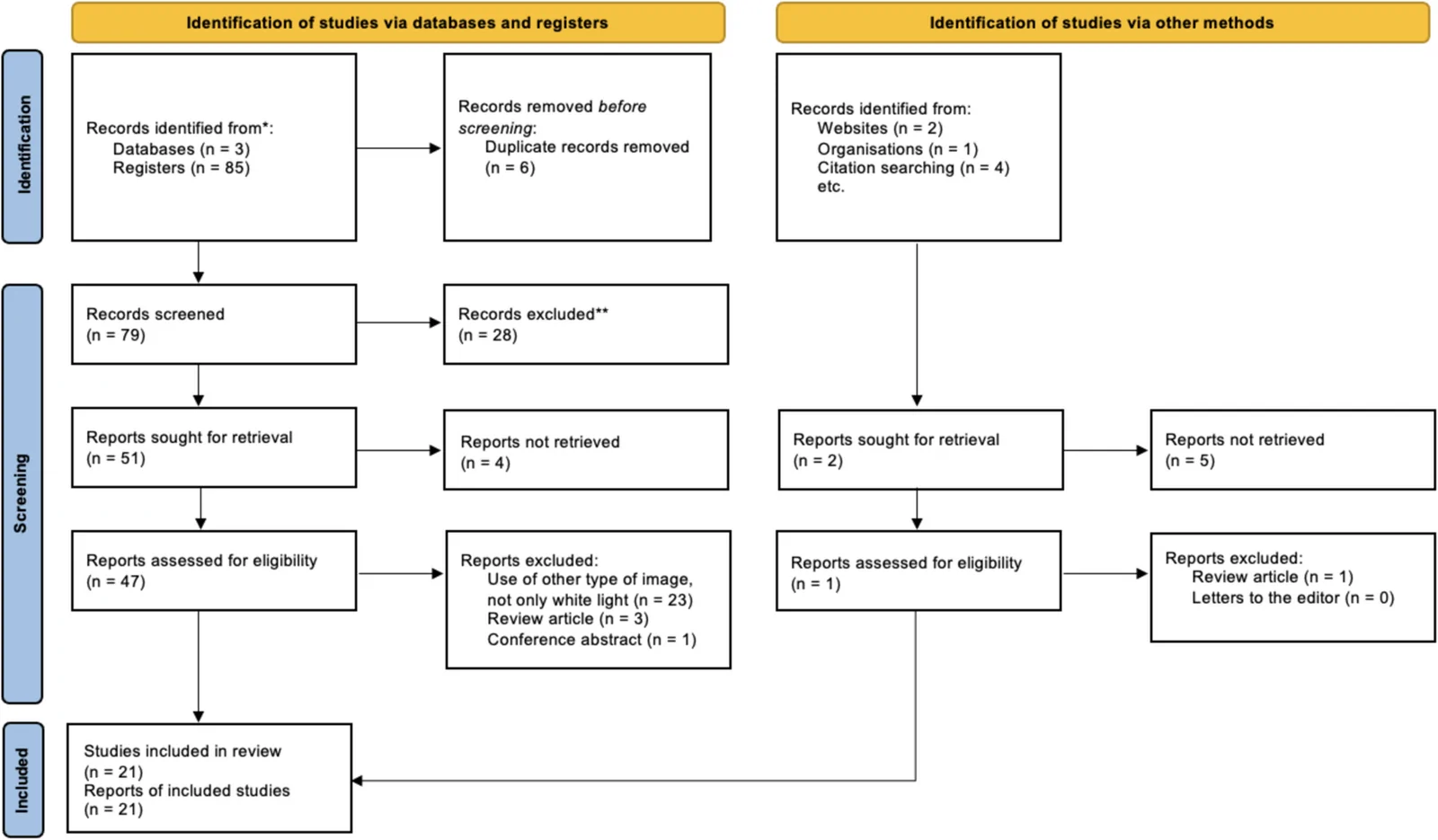
Flowchart of the bibliographic search and study selection process

The exhaustive search provided a total of 85 articles without any restrictions from three different databases (Web of Science, PubMed and Scopus). After the elimination of duplicate records, a total of 79 articles were obtained. After the application of the exclusion criteria, a total of 47 articles from the 51 selected were evaluated, as 4 articles were eliminated as they were not retrievable. Of the 47 articles, 27 were excluded due to different reasons, with a total of 20 articles used in the systematic review, with final number increasing to 21 after the inclusion of the grey literature. Ultimately, the meta-analysis was performed on 12 articles. The study selection process, as recommended by the PRISMA 2020 declaration [52], is detailed in Fig. 1.

### Risk of bias assessment

After the application of the QUADAS-C tool [50] to the 21 articles, it was observed that the risk of bias was high in 11, medium in 2, low in 6, and not clear in 2, as shown in supplementary material Table 2.

### Characteristics of the studies

Almost all the studies were conducted in the last 5 years, except for the study by Maghsoudi et al. [45], which was performed in 2013, as shown in Tables 3, 4 and 5 in the supplementary material, and of the 21 articles included in the study, 12 of them (57%) were conducted in the Asia.

The different types of models and baseline models are shown in Table 1, except for the articles by Fu et al. [7] and Maghsoudi et al. [45], which did not specify the type of AI utilized.

In the articles that mentioned the best Deep learning model, two concluded that the best was VGG19 [40, 41], one that it was DenseNet 169 [53], another that it was HRNet w18 [54], DenseNet201 [55], or U-Net [36], and lastly, the use of YOLOv4 [56].

With respect to the number of images, 12 of the studies included more than 1000 images, with the most being 5775 images. Only one included less than 150 images.

The images were captured with a smartphone or single-lens reflex digital camera. Of the 21 articles, 11 included used a histological control as the reference standard for lesion classification, whereas a smaller number relied primarily on expert clinical assessment or photographic annotation. Consequently, the interpretation of sensitivity and specificity values should be performed cautiously, as diagnostic performance may refer either to definitive pathological diagnosis or to clinician-based visual classification [7, 8, 29, 36, 48, 53, 54, 56–59].

**Fig. 2:**
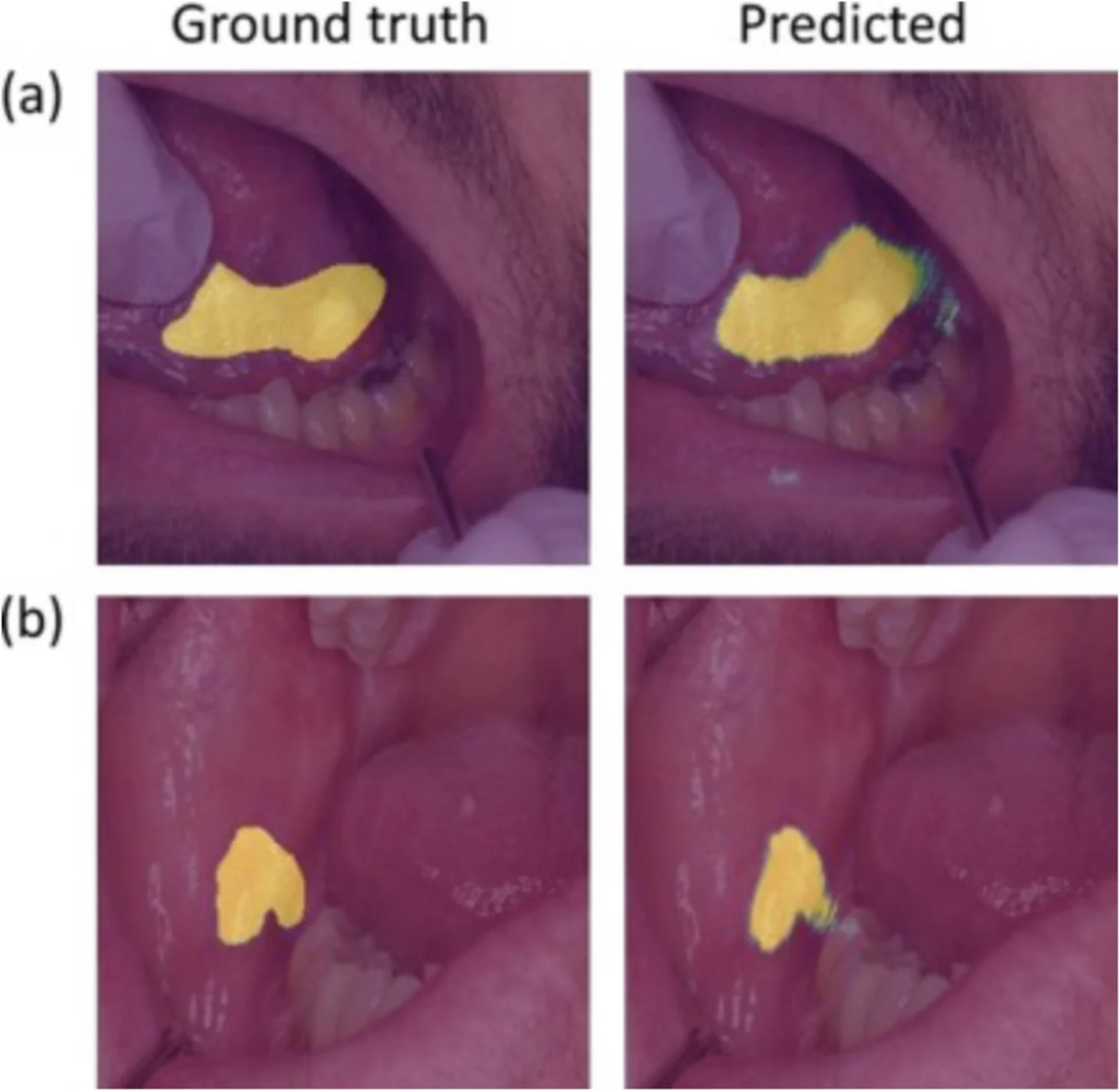
Example of the expert mask from two original photographs (left) and the generated (right) using U-Net [36]

In 16 studies, every part of the oral mucosa was photographed [7, 8, 32, 33, 41–43, 45, 53–60], in 2 only the buccal mucosa [40, 61], in another 2 only the tongue [29, 48], while one did not specify it [45] (Fig. 2).

Carcinomas and OPMD were photographed in 13 articles [7, 8, 29, 32, 41, 43, 48, 54, 55, 57–61], and in 8 articles [36, 40, 42, 45, 53, 56], only the OPMS were photographed.

With respect to the sensitivity and specificity, the highest sensitivity values were found in CNNmeta for the diagnosis of cancer (100%) and suspicious lesion (96%) [8]; googleNet Inception V3 for the detection of lichen planus (100%) [42], DenseNet121 (in 1 article 85% [55] and 100% in another [56]), DenseNet201 (85%) [55], SSD for cancer (93.9%) [29], DenseNet169 for cancer (99%), for oral potentially malignant disorders (95%), ResNet-101 for cancer (92%), for potentially malignant lesions (97%) [53], as shown in supplementary material Table 6. The highest specificity values were found with OCNN (81%) for cancer and OPMD (73%) [8], VGG-19 (90.5%), VGG-19 + GAIN (90.5%) [41], googleNet Inception V3 for lichen planus (97%) [42], DenseNet121 (100%) [55] and another 90% [56]), DenseNet201 (85%) [55], ResNet-50 (91.67%) [56], YOLOv8 for OSCC (98%) and OPMD (96%) [57] and DenseNet169 for cancer (99%) and for potentially malignant lesions (97%) [53]. Only two articles included values such as positive predictive value (PPV) or negative predictive value (NPV) [8, 29]. The remaining values found in the different studies are shown in supplementary material Table 6.

Posteriorly, a bivariate random effects meta-analysis was performed (Reitsma model), combining sensitivity and specificity of *N* = 12 studies, to analyze oral cancer, on the one hand, and OPMD on the other.

**Fig. 3:**
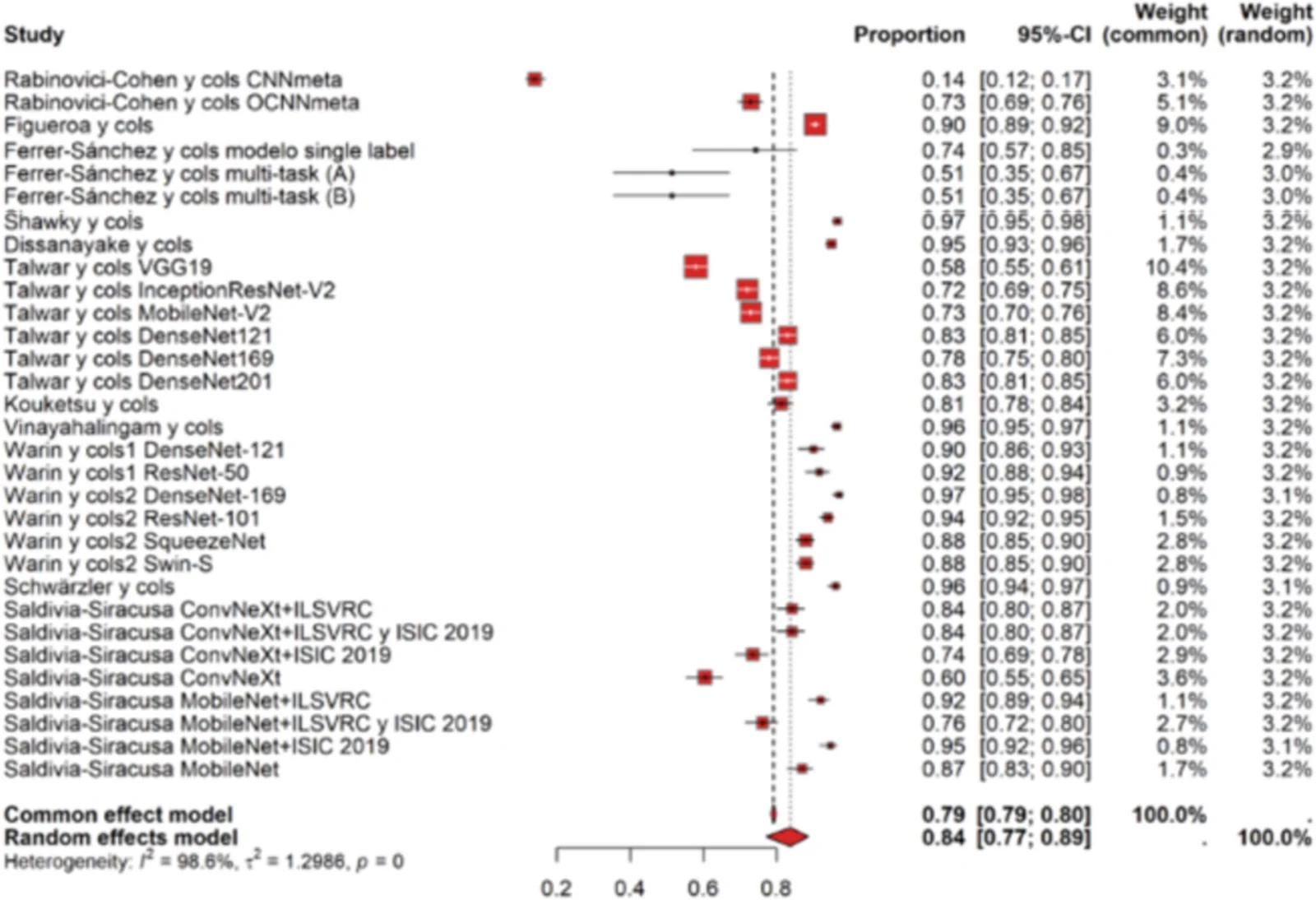
Forest specificity OPMD

**Fig. 4:**
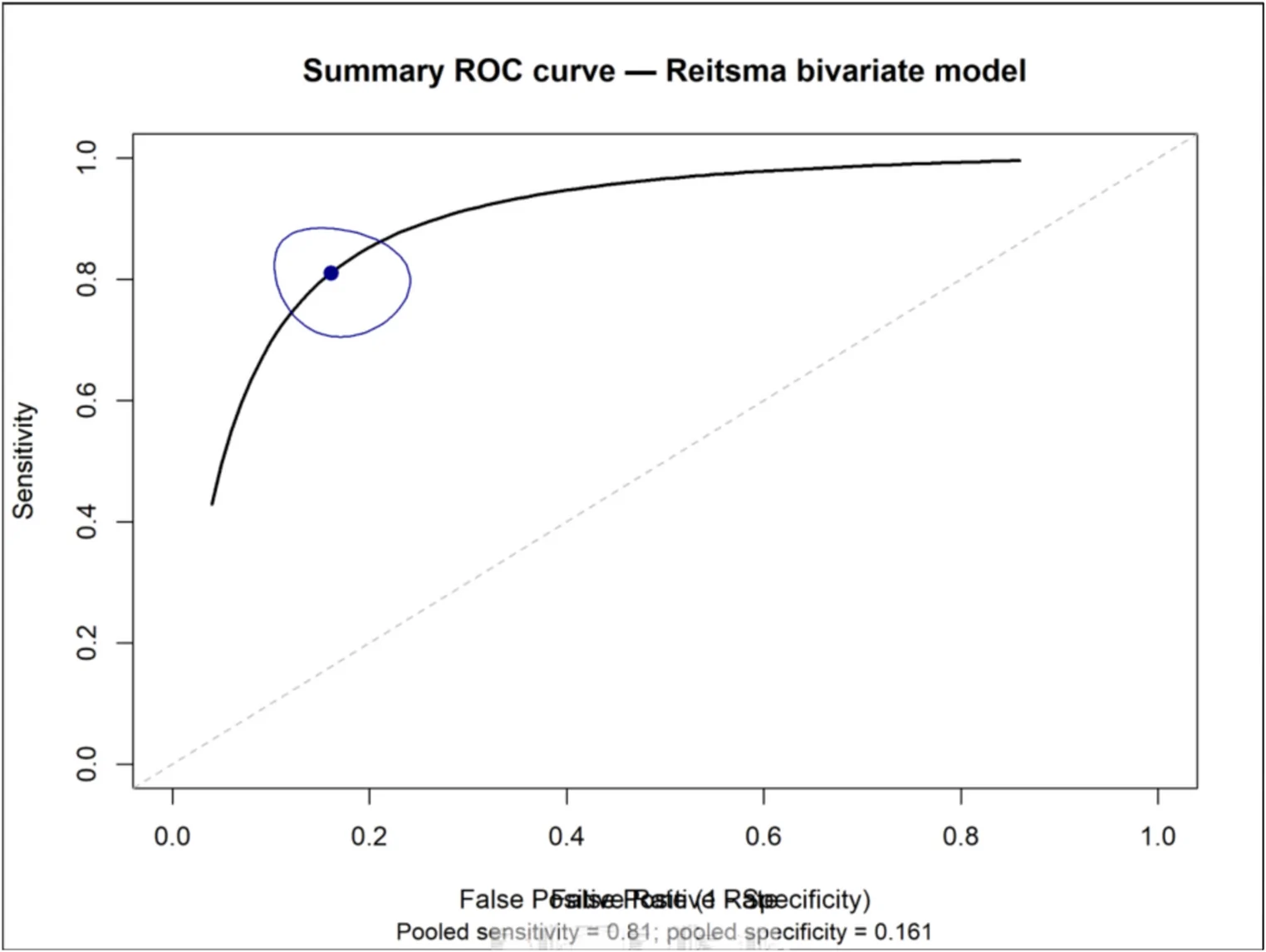
SROC OPMD

With respect to OPMD, the grouped sensitivity was 0.81 (95% CI 0.728–0.872) and the grouped specificity 0.161 (95% CI, 0.113–0.223). A very high heterogeneity was observed between studies (I² ≈ 98–99%, τ² in the Fig. 3 ≈ 1.3–2.1, *p* < 0.001). The SROC curve placed the summary point in a zone of high sensitivity and low specificity, with a wide prediction region as it can see in Fig. 4.

**Fig. 5:**
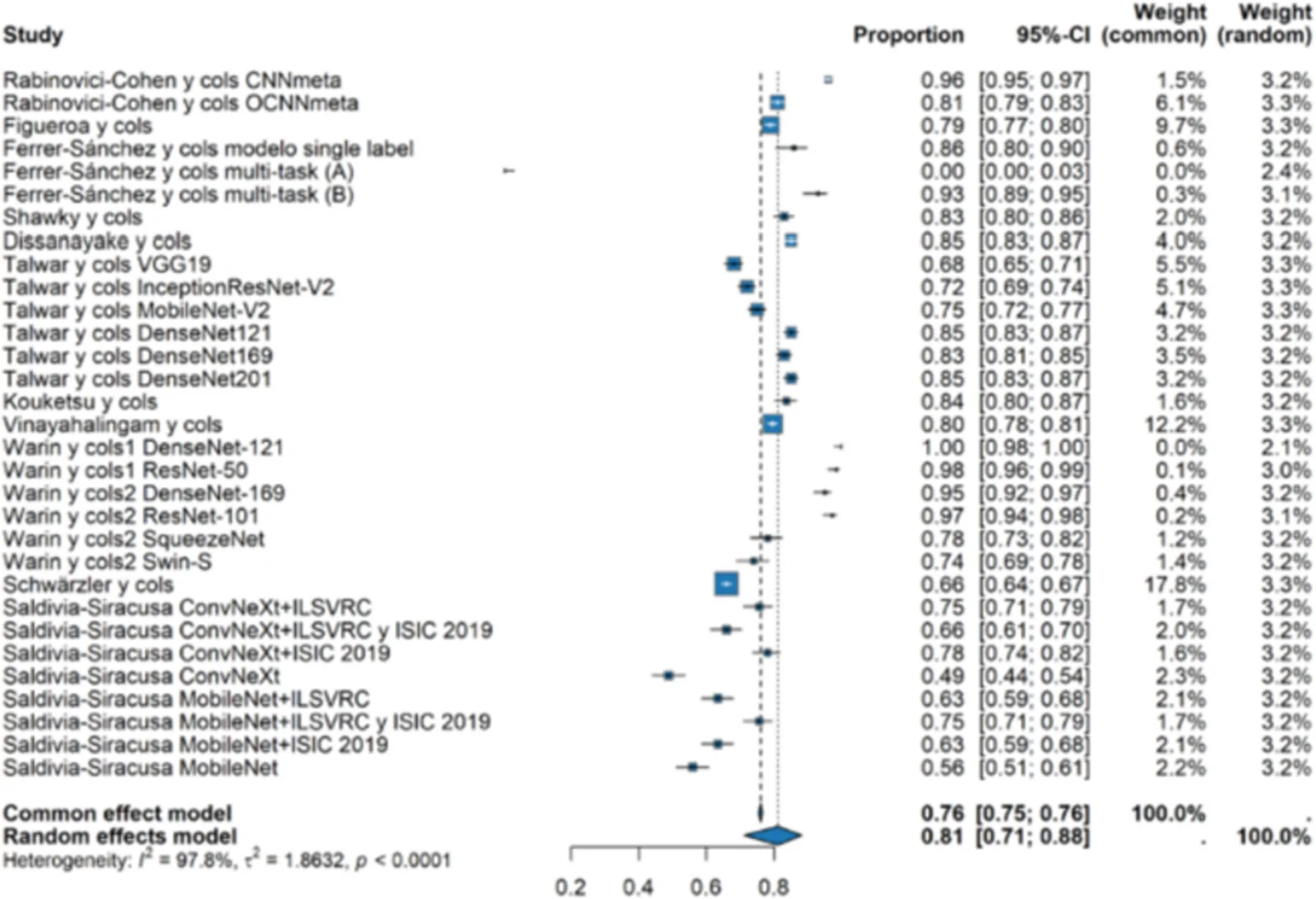
Forest sensitivity OPMD

The grouped results indicate that on average, the algorithms evaluated were sensitive for detecting OPMD (≈ 81%) (Fig. 5) but presented a low grouped specificity (≈ 16%), which would result in a high number of false positives if used as the sole test. The heterogeneity between studies was very large, so that the grouped estimates must be interpreted with caution.

**Fig. 6:**
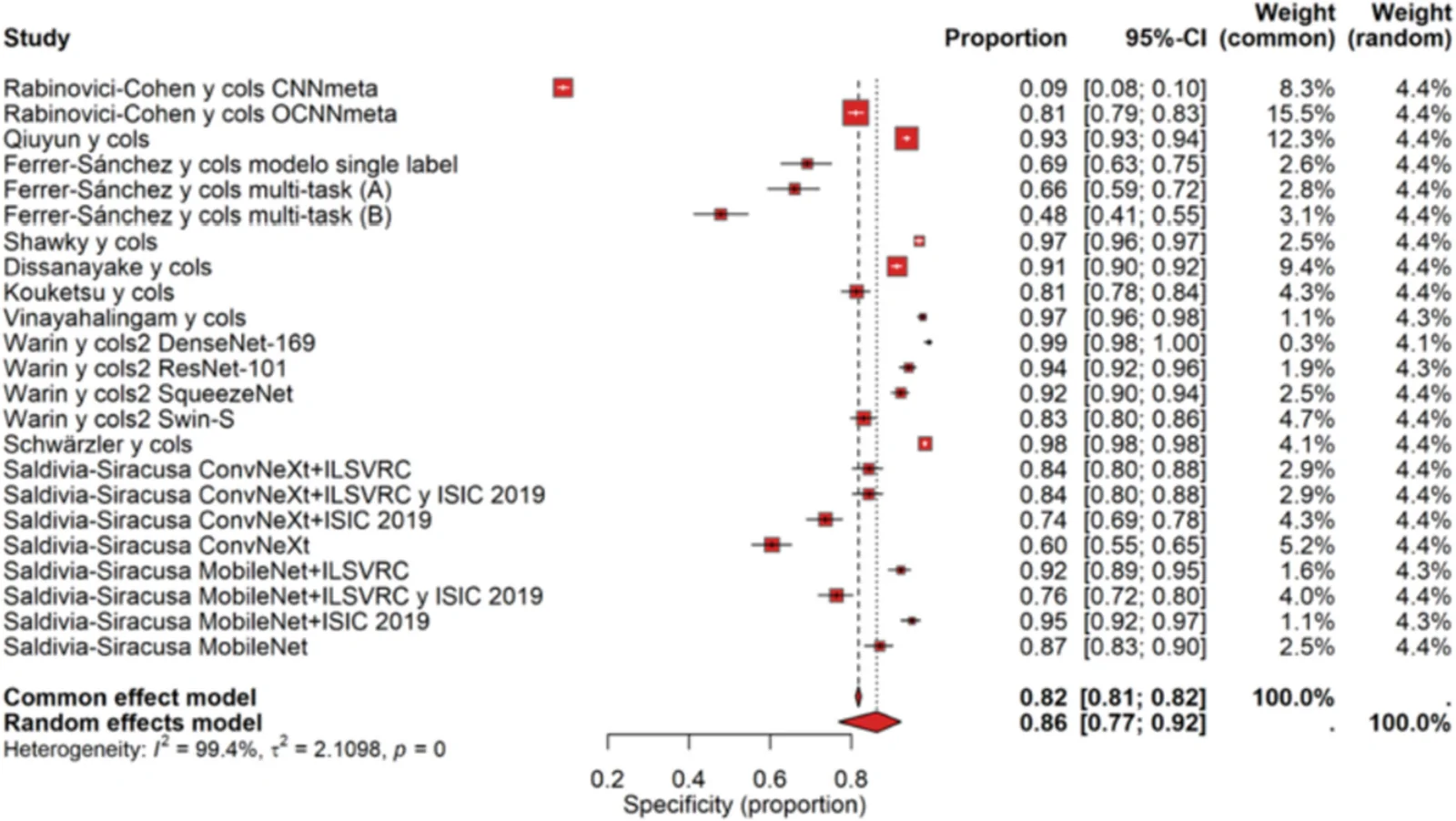
Forest specificity cancer

**Fig. 7:**
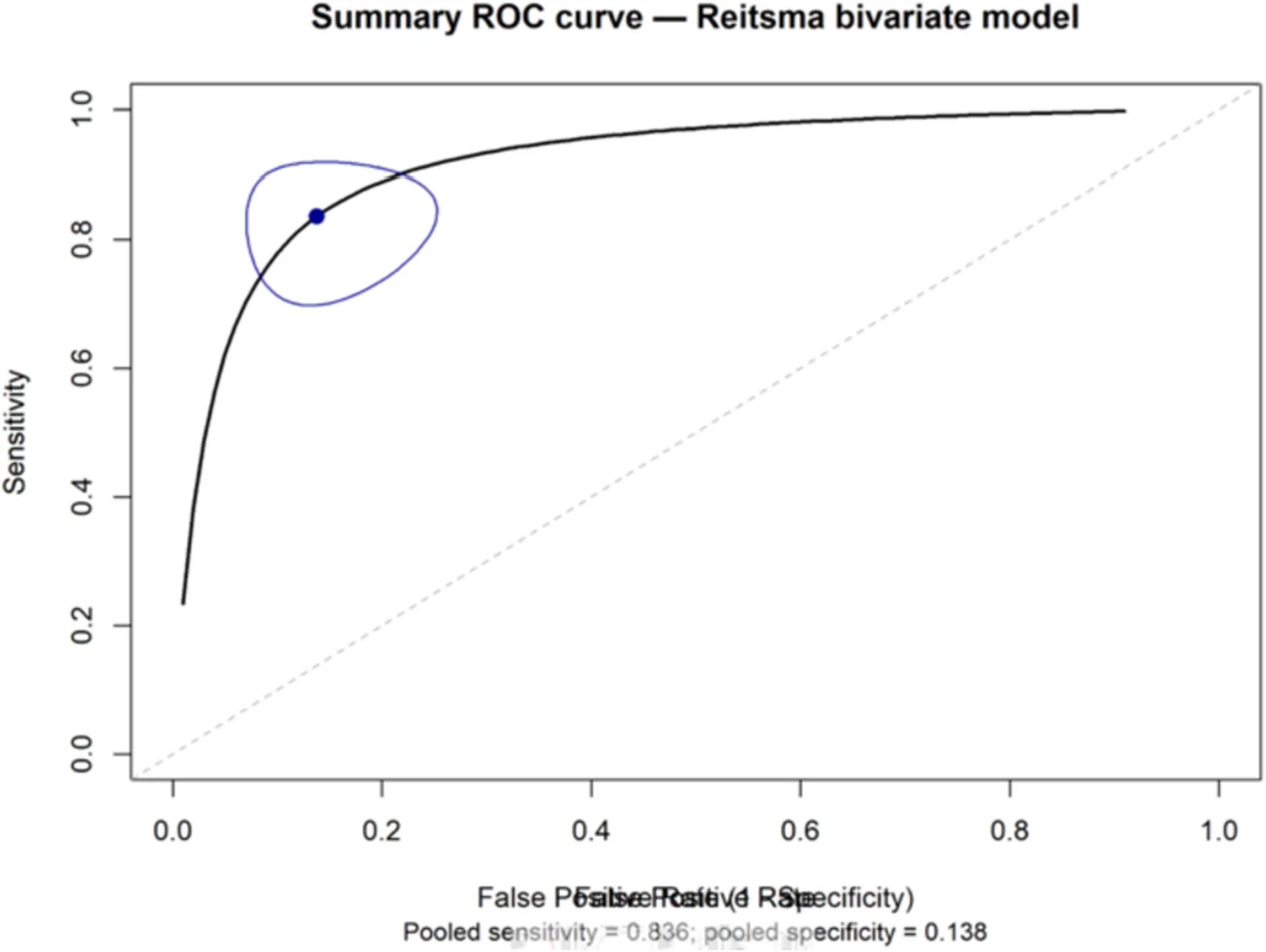
SROC cancer

**Fig. 8:**
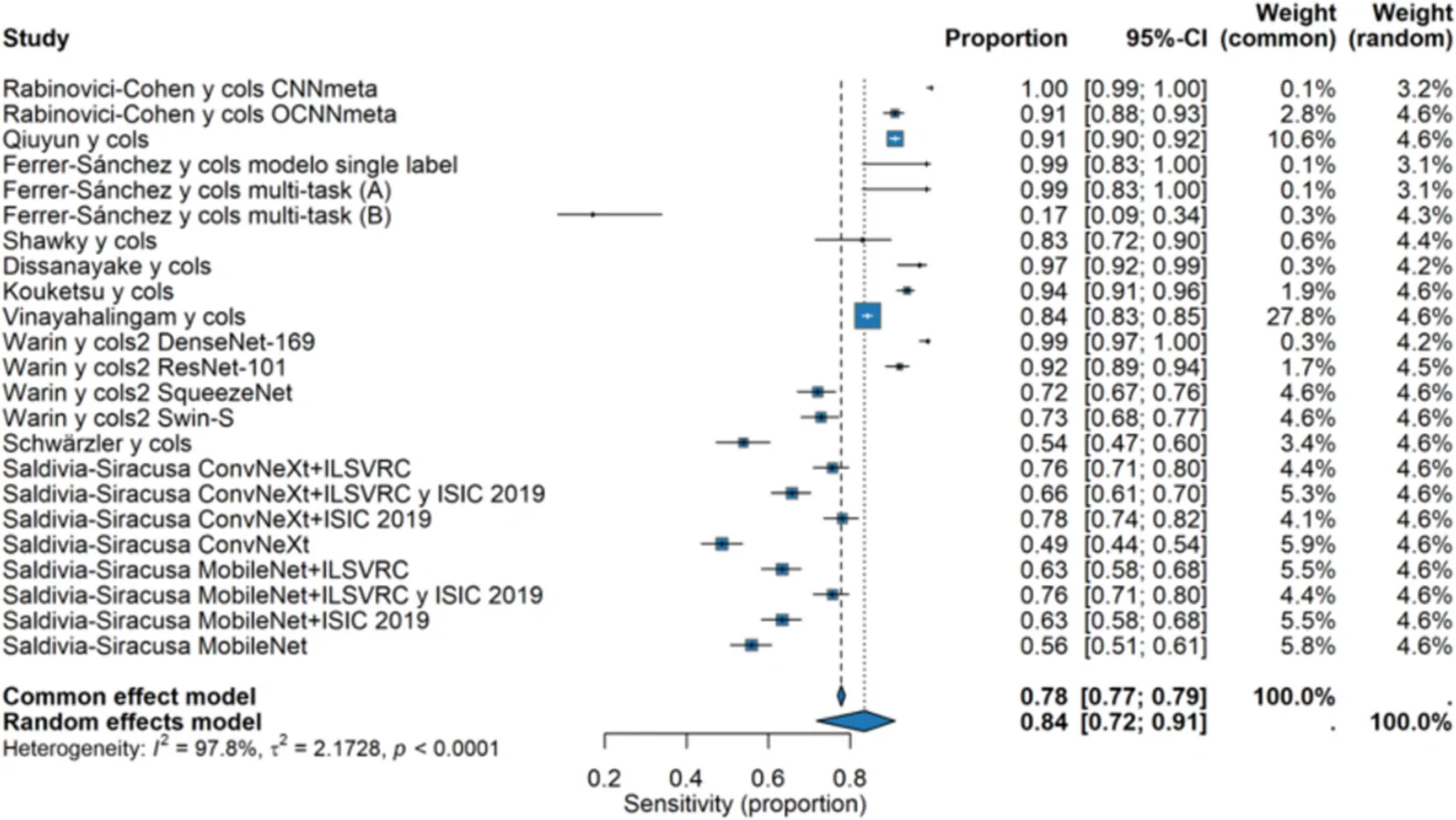
Forest sensitivity cancer

With respect to oral cancer, the combined sensitivity was 0.836 (95% CI: 0.730–0.906) and the combined specificity was 0.138 (95% CI: 0.081–0.226). The diagnostic odds ratio (DOR) was 0.814 (95% CI: 0.329–2.012). The area under the SROC curve was high (AUC = 0.994) (Fig. 7). However, a very large heterogeneity was observed between the studies (sensitivity I² ≈ 97–98%, specificity I² ≈ 99%), which indicates a substantial variability in performance between studies (Figs. 6, 7, and 8).

The combined estimations indicate that the algorithms were, on average, sensitive enough to detect oral cancer (≈ 84%), but with a low combined specificity (≈ 14%). In practice, this combination would lead to many false positives if the algorithms were used as an independent diagnosis. The DOR did not provide proof of a coherent discrimination between the studies (the CI included a value of 1), and the extremely high heterogeneity indicated that the combined averages hid large differences in the performance at the level of the study. The AUC estimated from the SROC seemed high, but this must be interpreted with caution, as the AUC could be determined by a subset of studies and by differences in the thresholds; in sets of heterogeneous data, it is possible for the AUC to not reflect the clinical operating characteristics.

As shown in Table 7 of the supplementary material, considerable heterogeneity was observed regarding model training strategies and hyperparameter selection across the included studies. Frequently reported parameters included input image sizes ranging from 224 × 224 to 300 × 300 pixels, transfer learning approaches, data augmentation techniques, and optimization algorithms such as Adam and stochastic gradient descent. However, several studies incompletely reported training configurations, limiting reproducibility and interstudy comparability.

## Discussion

Adjunctive diagnostic strategies have the potential to enhance early detection of OPMD and oral cancer, which is a key determinant of patient survival. The application of AI in this context offers a promising and innovative approach, enabling rapid analysis and high-throughput interpretation of clinical and imaging data, allowing its application to different areas of medicine offers a novel approach for OPMD screening and enables the diagnosis of oral cancer, demonstrating notable advantages with respect to the speed of the response and the high efficiency. The present systematic review and meta-analysis evaluated the diagnostic performance of deep learning algorithms for the detection of oral potentially malignant disorders (OPMDs) and oral cancer using conventional white-light photographic images. A total of 21 studies were included in the qualitative synthesis and 12 in the quantitative analysis. The pooled estimates demonstrated that deep learning models achieved acceptable sensitivity for the detection of both OPMDs (81%) and oral cancer (83.6%), supporting their potential role as adjunctive screening tools.

In the present study, the most frequently employed deep learning architectures were ResNet [8, 32, 41, 54, 56, 58] and VGG19 [41, 43, 44, 48, 55, 58], reflecting their widespread adoption in medical image analysis. In contrast, some studies [7, 59] opted to design deep learning algorithms specifically for the automated detection of oral cancer using intraoral photographs. These tailored models were capable of simultaneously localizing the lesion and categorizing it into distinct disease classes in a single step, representing an advancement over earlier two-stage approaches, such as those implemented by Maghsoudi et al. [45], Keser et al. [42], and Song et al. [44], which separated lesion detection from classification.

Jubair et al. [48] implemented a novel approach using the EfficientNet-B0 architecture, a lightweight transfer learning model known for achieving high accuracy on large-scale datasets while requiring fewer computational resources compared with traditional CNNs such as VGG19 and ResNet101. While no other studies included in this review employed EfficientNet-B0, VGG19 and ResNet101 remained the most commonly used architectures. The authors highlighted the potential of EfficientNet-B0 for deployment in mobile applications, offering a cost-effective, practical, and widely accessible tool for the early detection of oral cancer.

Rabinovici-Cohen et al. [8] evaluated standard and optimized convolutional neural networks, specifically ResNet18 and ResNet50, demonstrating that models pre-trained on clinical images outperformed those initialized with generic ImageNet weights, although diagnostic accuracy for cancer detection was slightly lower than for OPMD identification. Warin et al. [56] reported similar findings with ResNet50 and DenseNet-121, reporting high diagnostic accuracy with an AUC of 95% for both architectures when distinguishing lesions from normal mucosa. Their findings suggested that optimized CNNs may outperform other deep learning models, such as YOLOv4, in the detection of OPMD from intraoral images. Talwar et al. [55] also supported the idea that DenseNet, utilized by many studies included in the present review [53–56], offered many advantages, such as the mitigation of the vanishing gradient problem, the compatibility with the propagation and reutilization of characteristics, and the substantial reduction in the number of parameters. These characteristics allow DenseNet to maximize network capacity, producing compact, easily trainable models with high parameter efficiency and strong predictive performance.

The study conducted by Saldivia-Siracusa [58], which analyzed 778 clinical images from 681 patients, the ConvNeXt architecture—a recently developed CNN with several enhancements over traditional models—demonstrated the highest performance. Rabinovici-Cohen et al. [8] evaluated ResNet18 and an optimized ResNet50 in a cohort of 1470 patients, finding that models pre-trained on clinical images outperformed those initialized with generic weights, although diagnostic accuracy was slightly lower for cancer detection compared with OPMD identification. Across the studies included in this review, the average dataset used to train deep learning models comprised approximately 1955 images, reflecting the variability in sample sizes and the need for larger, more diverse datasets to enhance generalizability.

The acquisition of intraoral images for AI-based analysis is commonly performed using digital cameras or smartphones, followed by computational processing for automated lesion detection and classification. Although photographic capture is generally rapid and straightforward, the quality and consistency of images can vary substantially depending on camera specifications, lighting conditions, and resolution, which may directly affect the performance of deep learning models. While the studies included in this review used both digital cameras and smartphones, smartphones appear particularly advantageous due to their affordability, portability, and widespread availability, making them a feasible option for screening in resource-limited settings. Nevertheless, standardization of image capture protocols is critical to minimize variability and ensure that models trained on heterogeneous datasets maintain high diagnostic accuracy and generalizability across diverse clinical environments [7, 48, 62].

There is no protocol that complies recommendations for image acquisition for use with artificial intelligence, but this review proposes some recommendations listed in Table 8 [8, 29, 32, 33, 36, 41–44, 48, 53–56, 58, 59, 61].

In all the articles included in the present systematic review and meta-analysis, sensitivity values between 40% and 100% were obtained, depending on the models used and the diagnosis, and specificity values between 73% and 97%, with these results being similar to those obtained in a study by García-Pola et al. in 2021 [63].

All the articles coincided in that to be able to safely and efficiently use automated methods based on artificial intelligence in clinical practice, it is necessary to validate these algorithms using a robust experimental configuration. This configuration must include a clinically-representative dataset and adequate evaluation metrics for its validation [42, 54, 62–66].

In the present systematic review, an exhaustive analysis was performed, including values such as the combined sensitivity for cancer (0.836) and OPMD (0.81) and the combined specificity for cancer (0.138) and OPMD (0.161) and AUC of cancer (0.994). The general DOR for AI-based screening for cancer in the oral cavity was determined to be 81.4 (AUC = 0.994), a value that differed from that obtained in the meta-analysis performed by Li et al. [67], which obtained a general DOR for OPMD and cancer of 49.30 (AUC = 0.877). However, the diagnostic sensitivity obtained by this research group was similar to the one found in the present study (0.905).

Classification and object detection models represent fundamentally different computer vision tasks and should therefore be interpreted separately. Classification algorithms typically analyze predefined regions of interest or cropped lesion images, which may simplify the diagnostic task and artificially improve performance metrics. In contrast, object detection frameworks such as YOLO or Faster R-CNN attempt to simultaneously localize and classify lesions within full clinical photographs, representing a more challenging but clinically applicable scenario.

When pre-processing the image, two main approaches were found, one that used the entire image, and another that delimited the region of interest (ROI). In the study conducted by Jurczyszyn et al. [68], the ROI was selected in the center of the 300 × 300 pixel lesion without reference healthy mucosa, using a reference ROI of the mucosa of the same lesion. For example, if the lesion was on the tongue, the reference ROI of the tongue was selected. This technique was used in other studies, such as those conducted by Ferrer-Sánchez et al. [36] and Shawky et al. [54].

Incomplete reporting of hyperparameters and training procedures represented a frequent methodological limitation among the included studies. Variables such as learning rate, augmentation strategy, optimizer selection, and epoch number may substantially influence model performance and reproducibility. Greater adherence to standardized AI reporting frameworks is therefore needed in future studies. The limited use of explainable artificial intelligence techniques, such as saliency maps or Grad-CAM visualization, may also hinder clinical interpretability and trust. Future AI systems should incorporate explainability tools capable of identifying the image regions contributing most strongly to diagnostic predictions.

## Limitations

Compared with AI applications in other areas of medicine, research on AI-based diagnosis of OPMD and oral cancer remains relatively scarce and heterogeneous, underscoring the need for further studies with robust methodological designs. Many of the included studies relied on relatively small datasets, excluded clinically complex or ambiguous cases, and often lacked external validation, which may limit the generalizability and real-world applicability of the models. Furthermore, variability in image acquisition protocols, lighting conditions, and resolution introduces additional sources of heterogeneity that can impact diagnostic performance. These limitations highlight the necessity for standardized data collection, larger and more diverse datasets, and prospective validation to fully establish the reliability and clinical utility of AI-based approaches in oral cancer.

## Conclusions

Artificial intelligence demonstrates promising potential as a supportive tool for the early detection of oral cancer and oral potentially malignant disorders (OPMDs) through the analysis of clinical white-light images. The findings of this systematic review and meta-analysis suggest that deep learning models can achieve high diagnostic accuracy when applied to digital photographs, supporting their role as an adjunct to, rather than a replacement for, conventional clinical assessment. However, the included studies exhibited considerable heterogeneity regarding imaging protocols, datasets, AI architectures, validation strategies, and reference standards, with many studies also presenting a high risk of bias. These methodological limitations restrict the interpretability, reproducibility, and generalizability of the reported diagnostic performance.

Despite these limitations, AI-based analysis of clinical images remains a promising approach due to the accessibility and feasibility of image acquisition during routine clinical practice, particularly in low-resource and telemedicine settings. Future research should prioritize standardized image acquisition protocols, histopathologically validated datasets, external multicenter validation, transparent reporting of AI methodologies and hyperparameters, and adherence to established AI reporting guidelines to facilitate reliable clinical implementation.

## Supplementary information

Below is the link to the electronic supplementary material.

Supplementary Material 1

## Data Availability

No datasets were generated or analysed during the current study.
