# Supplementary material for: Detection of oral potentially malignant disorders and oral cancer with white light through deep learning: a systematic review and meta-analysis
Source: Clin Oral Investig. 2026 Aug 26;30(9):417. doi: 10.1007/s00784-026-07058-5 (PMC13518493; doi:10.1007/s00784-026-07058-5)
Supplement: Supplementary file 1 [file 784_2026_7058_MOESM1_ESM.docx]

SUPLEMMENTARY MATERIAL

**Table 1**. Search strategy in PubMed, Web of Science, and Scopus.

SEARCH ENGINES IN PubMed, Web of Science AND Scopus

PubMed

"Convolutional Neural Network" OR "machine learning" OR "Support Vector Machine" OR "random forest" OR "artificial intelligence" AND "oral potentially malignant disorders" OR "oral premalignant lesions" OR "oral premalignant diseases" OR "oral premalignant conditions" AND "image"

Web of Science

(((((((((((((TI=(artificial intelligence)) OR TI=(machine learning)) OR TI=(deep learning)) OR TI=(neural network)) OR TI=(artificial neural network)) OR TI=(convolutional neural network)) OR TI=(generative adversarial network)) OR TI=(transfer learning)) AND TI=(oral potentially malignant disorder)) OR TI=(opmd)) OR TI=(oral precancerous)) OR TI=(oral premalignant)) OR TI=(oral cancer)) AND TS=(photo)

SCOPUS

(TITLE-ABS-KEY(artificial AND intelligence) OR TITLE-ABS-KEY(machine AND learning) OR TITLE-ABS-KEY(deep AND learning) OR TITLE-ABS-KEY(neural AND network) OR TITLE-ABS-KEY(artificial AND neural AND network) OR TITLE-ABS-KEY(convolutional AND neural AND network) OR TITLE-ABS-KEY(generative AND adversarial AND networks) OR TITLE-ABS-KEY(transfer AND learning) AND TITLE-ABS-KEY(oral AND potentially AND malignant AND disorders) OR TITLE-ABS-KEY(opmd) OR TITLE-ABS-KEY(oral AND cancer) AND TITLE-ABS-KEY(photo))

**Table 2.** QUADAS-C for the assessment methodological quality and risk of bias in the articles selected.

| ARTICLE | QUADAS-C | | |
| --- | --- | --- | --- |
|  | RISK OF BIAS | APPLICABILITY | AGREEMENT |
| Dissanayake et al. (2022) | HIGH | MEDIUM | LOW |
| Ferrer-Sánchez et al. (2022) | HIGH | HIGH | HIGH |
| Figueroa et al, (2022) | NOT CLEAR | HIGH | MEDIUM |
| Fu et al. (2020) | LOW | HIGH | HIGH |
| Jubair et al (2021) | HIGH | MEDIUM | LOW |
| Keser et al. (2022) | HIGH | LOW | LOW |
| Kouketsu et al. (2024) | HIGH | MEDIUM | HIGH |
| Maghsoudi et al. (2013) | MEDIUM | MEDIUM | MEDIUM |
| Rabinovici-Cohen et al. (2024) | LOW | HIGH | HIGH |
| Saldivia-Siracusa et al (2025) | LOW | MEDIUM | MEDIUM |
| Shawky et al. (2022) | HIGH | MEDIUM | MEDIUM |
| Schwärzler et al (2025) | HIGH | NOT CLEAR | HIGH |
| Song et al. (2021a) | NOT CLEAR | MEDIUM | MEDIUM |
| Song et al. (2021b) | HIGH | HIGH | HIGH |
| Song et al. (2021c) | MEDIUM | MEDIUM | MEDIUM |
| Talwar et al. (2023) | LOW | LOW | HIGH |
| Vinayahalingam et al. (2024) | LOW | HIGH | HIGH |
| Warin et al. (2022a) | HIGH | MEDIUM | MEDIUM |
| Warin et al (2022b) | HIGH | HIGH | HIGH |
| Wuttisarnwattana et al. (2024) | HIGH | HIGH | HIGH |
| Ye et al. (2024) | LOW | HIGH | HIGH |

**Table 3.** Characteristics of the study.

| ARTICLE | YEAR | COUNTRY | TYPE OF STUDY | DEEP LEARNING MODEL | N. IMAGES | TOOL | HISTOLOGIAL CONTROL |
| --- | --- | --- | --- | --- | --- | --- | --- |
| Rabinovici-Cohen et al. [8] | 2024 | Israel | Retrospective study | ResNet18 | 2398 | smartphone | Yes |
| Song et al. [40] | 2021 | India | Cross-sectional study | VGG-19 | 1979 | Smartphone | / |
| Figueroa et al. [41] | 2022 | India | Cross-sectional study | VGG-19 | 5200 | Digital camera | / |
| Song et al. [43] | 2022 | India | Cross-sectional study | U-NET | 492 | Digital camera | / |
| Jubair et al [48] | 2021 | Jordan | Cross-sectional study | EfficientNet-BO | 716 | Digital camera/ smartphone | Yes |
| Fu et al. [7] | 2020 | China | Retrospective study | / | 5775 | smartphone | Yes |
| Maghsoudi et al. [45] | 2013 | Iran | Cross-sectional study | / | 130 | / | / |
| Ferrer-Sánchez et al. [36] | 2022 | Spain | Retrospective study | U-NET | 261 | / | Yes |
| Ye et al. [59] | 2024 | China | Retrospective study | YOLOX | 1419 | Smartphone and digital camera | Yes |
| Shawky et al. [54] | 2024 | Egypt | Retrospective study | HRNet-W18 | 14[48] | Smartphone | Yes |
| Keser et al. [42] | 2022 | Turkey | Retrospective study | GoogleNet Inception | 137 | / | / |
| Song et al. [61] | 2021 | India | Cross-sectional study | VGG19 | 2417 | / | / |
| Dissanayake et al. [33] | 2024 | China | Cross-sectional study | YOLOX | 2271 | Smartphone and digital camera | / |
| Talwar et al. [55] | 2023 | India | Cross-sectional study | DenseNet201 | 2178 | smartphone | / |
| Wuttisarnwattana et al. [32] | 2024 | Thailand | Retrospective study | ResNet50 | 2591 | smartphone | / |
| Kouketsu et al. [29] | 2024 | Japan | Cross-sectional study | SSD | 1043 | Digital camera | Yes |
| Vinayahalingam et al. [60] | 2024 | Germany | Retrospective study | YOLOX | 4161 | / | / |
| Warin et al. [56] | 2022 | Thailand | Retrospective study | DenseNet-169 and ResNet | 600 | Digital camera | Yes |
| Warin et al. [53] | 2022 | Thailand | Retrospective study | Faster R-CNN | 980 | / | Yes |
| Schwärzler et al. [57] | 2025 | Germany | Retrospective study | YOLOv8 | 4079 | Digital camera | Yes |
| Saldivia-Siracusa et al. [58] | 2025 | Brazil | Retrospective study | MobileNetv2 | 778 | Smartphone and digital camera | Yes |

**Table 4**. Characteristics of the studies-continued.

| ARTICLES | LESION LOCATION | SENSITIVITY (Recall) | SPECIFICITY | ACCURACY |
| --- | --- | --- | --- | --- |
| Rabinovici-Cohen et al. [8] | Full mouth | CANCER: CNNmeta 100% OCNN 91%. SUSPICIOUS TASK: CNNmeta 96% OCNNmeta 81% | CANCER CNNmeta 9% OCNN 81% SUSPICIOUS TASK: CNNmeta 14% OCNNmeta 73% | CANCER CNNmeta 54% OCNN 86% SUSPICIOUS TASK: CNNmeta 55% OCNNmeta 77% |
| Song et al. [40] | Cheek mucosa | / | / | 85.1% |
| Figueroa et al. [41] | Full mouth | CANCER 78.9% | CANCER 90.5% | 84.84% |
| Song et al. [43] | Full mouth | / | / | 91% |
| Jubair et al [48] | Tongue | 86.7% | 84.5% | 83% |
| Fu et al. [7] | Full mouth | 91% | 93.5% | 92.3% |
| Maghsoudi et al. [45] | / | / | / | / |
| Ferrer-Sánchez et al. [36] | Full mouth | 100% OPMD 85.7% CANCER | 69.2% OPMD 74% CANCER | 74% OPMD |
| Ye et al. [59] | Full mouth | 91.11% CANCER | 95% CANCER | 78.38% |
| Shawky et al. [54] | Full mouth | 83% OPMD and CANCER | 96.6% OPMD and CANCER | 84.3% |
| Keser et al. [42] | Full mouth | / | / | / |
| Song et al. [61] | Cheek mucosa | CANCER 75% OPMD 82% | / | VGG19 81% |
| Dissanayake et al. [33] | Full mouth | 96.67% OPMD 85% CANCER | 91.11% OPMD 95% CANCER | 87.02% OPMD 78.38% CANCER |
| Talwar et al. [55] | Full mouth | VGG19: 68% ResNetV2 72% MobileNet-V2 75% DenseNet121 85% DenseNet169 83% DenseNet201 85% | VGG19: 58% ResNetV2 72% MobileNet-V2 73% DenseNet121 83% DenseNet169 78% DenseNet201 83% | VGG19: 69% ResNetV2 72% MobileNet-V2 75% DenseNet121 85% DenseNet169 84% DenseNet201 86% |
| Wuttisarnwattana et al. [32] | Full mouth | / | / | 87.5% |
| Kouketsu et al. [29] | Tongue | 93.9% OPMD 83.7% CANCER | 81.2% OPMD and CANCER | 81.7% OPMD 83% CANCER |
| Vinayahalingam et al. [60] | Full mouth | 84.3% OPMD 79.5% CANCER | 97.5% OPMD 96.5% CANCER | 93% OPMD 95.5% CANCER |
| Warin et al. [56] | Full mouth | DenseNet-121 100% ResNet-50 98.39% | DenseNet-121 90% ResNet-50 91.67% | DenseNet-121 91% ResNet-50 92% |
| Warin et al. [53] | Full mouth | DenseNet-169 99% OPMD 95% CANCER ResNet-101 92% OPMD 97% CANCER SqueezeNet 72% OPMD 78% CANCER Swin-S 73% OPMD 74% CANCER | DenseNet-169 99% OPMD 97% CANCER ResNet-101 94% OPMD 94% CANCER SqueezeNet 92% OPMD 88% CANCER Swin-S 83% OPMD 88% CANCER | DenseNet-169 98% ResNet-101 96% SqueezeNet 85% Swin-S 69% |
| Schwärzler et al. [57] | Full mouth | 54% OPMD 65.9% CANCER | 98%OPMD 96% CANCER | 62% OPMD 65% CANCER |
| Saldivia-Siracusa et al. [58] | Full mouth | ConvNext 75.6% OPMD and CANCER; MobileNet 56% OPMD and CANCER | ConvNext 84.2% OPMD and CANCER; MobileNet 86.8% OPMD and CANCER | ConvNext 75% OPMD and CANCER; MobileNet 79% OPMD and CANCER |

**Table 5.** Characteristics of the studies-continued.

| ARTICLES | F1-SCORE | AUC | PIXEL | ROI/WHOLE IMAGE |
| --- | --- | --- | --- | --- |
| Rabinovici-Cohen et al. [8] | CANCER CNNmeta 37% OCNN 69% SUSPICIOUS TASK: CNNmeta 52% OCNNmeta 68% | CANCER CNNmeta 92% OCNN 96% SUSPICIOUS TASK: CNNmeta 86% OCNNmeta 85% | / | / |
| Song et al. [40] | / | / | / | / |
| Figueroa et al. [41] | / | / | 20 |  |
| Song et al. [43] | / | / | 0.881 accuracy | / |
| Jubair et al [48] | / | 92.8% | 224x224 | ROI |
| Fu et al. [7] | / | 97% | / | / |
| Maghsoudi et al. [45] | / | / | / | / |
| Ferrer-Sánchez et al. [36] | / | / | 6.91% | ROI |
| Ye et al. [59] | / | / | / | / |
| Shawky et al. [54] | 83.6% | CANCER 94.6% OPMD 86% | 0.255-1.0 | ROI |
| Keser et al. [42] | / | / | / | / |
| Song et al. [61] | CANCER 84% OPMD 73% | 89% | / | / |
| Dissanayake et al. [33] | / | / | 224x224 | ROI |
| Talwar et al. [55] | VGG19 68%; InceptionResNet-V2 72%; MobileNet-V2 75%; DenseNet121 85%; DenseNet169 84%; Densenet201 86% | / | 224x224 | ROI |
| Wuttisarnwattana et al. [32] | / | / | / | / |
| Kouketsu et al. [29] | 81.2% OPMD and CANCER | 81.7% OPMD 83% CANCER | 300x300 | ROI |
| Vinayahalingam et al. [60] | 81.5% OPMD 85.2% CANCER | / | 74 per inch | ROI |
| Warin et al. [56] | DenseNet-121 90% ResNet-50 91.67% | DenseNet-121 91% ResNet-50 92% | 1920X1280 | / |
| Warin et al. [53] | CANCER: DenseNet-169 98%; ResNet-10194%; SqueezeNet 78%; Swin-S 71%; Faster R-CNN 87%; YOLOv5 87%; RetinaNet 89%; CenterNet2 76%  OPMDs: DenseNet-169 95%; ResNet-101 97%; SqueezeNet 77%; Swin-S 68%; Faster R-CNN 65%; YOLOv5 51%; RetinaNet 70%; CenterNet2 54% | CANCER: DenseNet-169 100%; ResNet-101 99%; SqueezeNet 88%; Swin-S 71%; Faster R-CNN 88%; YOLOv5 84%; RetinaNet 81%; CenterNet2 91%  OPMDs: DenseNet-169 98%; ResNet-101 97%; SqueezeNet 87%; Swin-S 80%; Faster R-CNN 64%; YOLOv5 34%; RetinaNet 55%; CenterNet2 58% | 4496x3000 to 1081x836 | ROI |
| Schwärzler et al. [57] | 98% OPMD 96% CANCER | 62% OPMD 65% CANCER | 640x640 | ROI |
| Saldivia-Siracusa et al. [58] | ConvNext 79.4% MobileNet 75.3% | ConvNext 86.3% MobileNet 85.3% | 224x224 | ROI |

**Table 7.** AI training characteristics and hyperparameters.

| ARTICLES | Input size | Optimizer | Epochs | Bacht size | Augmentation |
| --- | --- | --- | --- | --- | --- |
| Rabinovici-Cohen et al. [8] | 224x224 300x300 | Adam optimezer | 30 | 32 | rotation, scaling, flipping, and the addition of Gaussian noise. |
| Song et al. [40] | / | Adam optimezer | 300 | 32 | yes |
| Figueroa et al. [41] | / | 20 | 55 | 8 | Random rotations, reflections, translation, and padded any blank pixels at the edges of the image with RGB values of [128, 128, 128] |
| Song et al. [43] | / | Adam optimezer | 300 | 16 | horizontal/vertical flipping,rotation, zoom, brightness adjustment, and gamma correction |
| Jubair et al [48] | 224x224 | Adam optimezer | 5 | 32 | / |
| Fu et al. [7] | / | / | / | / | scaling, rotation, horizontal ﬂipping and adjustment of the saturation and exposure |
| Maghsoudi et al. [45] | / | / | / | / | / |
| Ferrer-Sánchez et al. [36] | / | / | / | / | random displacements, zooms,rotations, etc. |
| Ye et al. [59] | / | yolox | / | / | yes |
| Shawky et al. [54] | 0,255-1.0 | / | 100 | 24 | yes |
| Keser et al. [42] | / | / | 2000 | / | / |
| Song et al. [61] | / | Adam optimezer | 300 | 32 | yes |
| Dissanayake et al. [33] | 224x224 | Adam optimizer | 200 | 64 | TrivialAugmentation |
| Talwar et al. [55] | 224X224 | Adam optimizer | 50 | 16 | / |
| Wuttisarnwattana et al. [32] | / | / | / | / | / |
| Kouketsu et al. [29] | / | / | 300 | / | yes |
| Vinayahalingam et al. [60] | / | AdamW optimizer | 16 and 22 | 4 | yes |
| Warin et al. [56] | 224x224 | / | 100 | 32 | Keras Image Data Generator |
| Warin et al. [53] | 224x224 | / | 43 | 32 | Keras Image Data Generator |
| Schwärzler et al. [57] | / | Adam optimezer | 300 | 32 | rotation, flipping, scaling, and mosaicking in every epoch. |
| Saldivia-Siracusa et al. [58] | / | Adam optimezer | 250 | / | random translations, rotations, mirroring,  and shearing |

**Table 6.** Different types of model and base model that were used in the different selected articles

| ARTÍCULOS | **1** | **2** | **3** | **4** | **5** | **6** | **7** | **8** | **9** | **10** | **11** | **12** | **13** | **14** | **15** | **16** | **17** | **18** | **19** |
| --- | --- | --- | --- | --- | --- | --- | --- | --- | --- | --- | --- | --- | --- | --- | --- | --- | --- | --- | --- |
| **RES-NET 50** | X |  | X |  |  |  |  | X |  |  |  |  | X |  |  |  | X |  | X |
| **VGG19** |  | x | X |  | X |  |  |  |  | X |  | X |  |  |  |  |  |  | X |
| **DenseNet169** |  |  |  |  |  |  |  | X |  |  |  | X |  |  |  | X |  |  |  |
| **DenseNet121** |  |  |  |  |  |  |  |  |  |  |  | X |  |  |  |  | X |  |  |
| **RES-NETv2** |  |  | X |  |  |  |  |  |  |  |  |  |  |  |  |  |  |  | X |
| **U-Net arquitecture** |  |  |  | X |  |  |  |  |  |  |  |  |  |  |  |  |  |  |  |
| **Faster R-CNN** | x |  |  |  |  |  |  |  |  |  |  |  |  |  |  |  | X |  |  |
| **YOLOX** |  |  |  |  |  |  | X |  |  |  | X |  |  |  | X |  |  |  |  |
| **DenseNet201** |  |  |  |  |  |  |  |  |  |  |  | X |  |  |  |  |  |  |  |
| **SqueezenET** |  |  |  |  |  |  |  |  |  |  |  |  |  |  |  | X |  |  |  |
| **Swin-S** |  |  |  |  |  |  |  |  |  |  |  |  |  |  |  | X |  |  |  |
| **YOLOv5** |  |  |  |  |  |  |  |  |  |  |  |  |  |  |  | X |  |  |  |
| **RetinaNet** |  |  |  |  |  |  |  |  |  |  |  |  |  |  |  | X |  |  |  |
| **CenterNet2** |  |  |  |  |  |  |  |  |  |  |  |  |  |  |  | X |  |  |  |
| **YOLOv4** |  |  |  |  |  |  |  |  |  |  |  |  |  |  |  |  | X |  |  |
| **Mask R-CNN** |  |  |  |  |  |  |  |  |  |  |  |  |  |  |  |  |  |  |  |
| **MobileNet v2** |  |  |  |  |  |  |  |  |  | X |  |  |  |  |  |  |  |  |  |
| **GoogleNet inception** |  |  |  |  |  |  |  |  | X |  |  | X |  |  |  |  |  |  |  |
| **VGG16** |  |  |  |  |  |  |  | X |  |  |  |  |  |  |  |  |  |  | X |
| **HRNet W18** |  |  |  |  |  |  |  | X |  |  |  |  |  |  |  |  |  |  |  |
| **ResNet18** | x |  |  |  |  |  |  |  |  |  |  |  |  |  |  |  |  |  | X |
| **SSD** |  |  |  |  |  |  |  |  |  |  |  |  |  | X |  |  |  |  |  |
| **UNET** |  |  |  |  |  | X |  |  |  |  |  |  |  |  |  |  |  |  |  |
| **MLP** |  |  |  |  |  |  |  |  |  |  |  |  |  |  |  | X |  |  |  |
| **ResNet101** |  |  |  |  | X |  |  |  |  |  |  |  |  |  |  |  |  |  |  |
| **EfficientNet** |  |  |  |  | X |  |  |  |  |  |  |  |  |  |  |  |  |  |  |
| **YOLOv8** |  |  |  |  |  |  |  |  |  |  |  |  |  |  |  |  |  | X |  |

Articles: 1:Rabinovici-Cohen et al [8], 2: Song et al [40] 3: Figueroa et al [41] 4: Song et al [43], 5: Jubair et al [48], 6: Ferrer-Sánchez et al [36] 7: Ye et al [59] , 8: Shawky et al [54] 9: Keser et al [42], 10: Song et al [61], 11: Dissanayake et al [33], 12: Talwar et al [55], 13: Wuttisarnwattana et al [32] 14: Kouketsu et al [29] 15: Vinayahalingam et al [60], 16: Warin et al [56], 17: Warin et al [53], 18: Schwärzler et al [57] and 19: Saldiva-Siracusa [58]

**Table 8**. Recommended image acquisition protocol.

| **TECHNICAL FACTOR** | **RECOMMENDATION** |
| --- | --- |
| CAMERA | Smartphone or Digital single-lens reflex camera with autofocus and macro/close focus |
| RESOLUTION | >= 224x224 ROI  >= 8-12MP |
| BACKGROUND | Include adjacent healthy mucosa |
| DATASET SIZE | Preferably >1000 images |
| LIGHTING | Uniform white LED lighting, stable color temperature (5000-6500K), avoid harsh direct flash and shadows |
